# A survey of the prevalence of smoking and smoking cessation advice received by in patients in two teaching hospitals in Ireland

**DOI:** 10.1186/1753-6561-9-S1-A54

**Published:** 2015-01-14

**Authors:** AL Ohakim, B Jafar, C O’Bryne, NG McElvaney

**Affiliations:** 1 Royal College of Surgeons in Ireland, Dublin 2, Ireland; 2 Respiratory Research Division, Department of Medicine, Royal College of Surgeons in Ireland

## Background

Brief cessation advice from healthcare professionals significantly increases the likelihood of patients quitting smoking, yet patients are not routinely provided with this advice. Smoke-free Brief cessation advice from healthcare professionals significantly increases the likelihood of patients quitting smoking, yet patients are not routinely provided with this advice. Smoke-free hospital policies aim to protect individuals from the adverse effects of smoking, however it is not clear if it encourages patients to quit or doctors to give smoking cessation advice. The aim was to determine the prevalence of smoking and cessation advice received by in- patients in two teaching hospitals in Ireland, which have smoke-free hospital policies. We also compared data from one hospital from before and after the policy implementation.

## Methods

This study surveyed 466 eligible in-patients, 260 in Beaumont hospital and 206 in Connolly hospital; assessing their smoking habits, and advice they received from healthcare professionals on smoking cessation.

## Results

Smoking prevalence was higher in Connolly (28%) compared to Beaumont (17%). 26% of current smokers in Connolly had received smoking cessation advice compared to 51% in Beaumont. The before-and-after analysis of Beaumont (2011 vs. 2013) showed a reduction in smoking prevalence and an increase in cessation advice.

## Conclusions

Smoke-free hospital policies play a role in decreasing the prevalence of in-patient smokers.

